# A preliminary study on the clinical characteristics of lacrimal duct obstruction in patients with a history of anterior uveitis

**DOI:** 10.3389/fmed.2025.1630425

**Published:** 2025-07-04

**Authors:** Peng Wang, Hai Tao, Fei Wang, Fang Bai, Xibin Zhou, Lihua Wang, Chuan Liu, YiFei Huang

**Affiliations:** ^1^Medical School of Chinese PLA, Beijing, China; ^2^Department of Ophthalmology, The Third Medical Center, Chinese PLA General Hospital, Beijing, China

**Keywords:** lacrimal duct obstruction, anterior uveitis, clinical characteristics, diagnosis and therapy, surgery method, therapeutic effect

## Abstract

**Aim:**

This study aimed to investigate the clinical features of lacrimal duct obstruction (LDO) in patients with a history of anterior uveitis (AU) and explore preventive strategies by analyzing their demographic, anatomical, and surgical outcome profiles.

**Methods:**

We retrospectively reviewed 40 LDO patients (58 eyes) with a history of AU, treated between 2020 and 2024, comparing them to non-AU LDO controls. Data included demographics, AU–epiphora temporal relationships, obstruction characteristics (location, extent, and adhesions), and surgical outcomes. Statistical analysis evaluated differences between groups.

**Results:**

The mean age was 49.88 ± 10.18 years, with 30 women (75%) and 10 men (25%). Proximal lacrimal canalicular obstruction (<4 mm from lacrimal punctum) occurred in 32 cases (80%), comprising 16 cases of lacrimal punctal atresia, 6 cases of proximal lacrimal canalicular obstruction, and 10 cases with a combination of both conditions. Additional cases included one (2.5%) middle lacrimal canalicular obstruction and two (5%) distal lacrimal canalicular obstructions. In total, three (7.5%) had proximal and distal lacrimal canalicular obstructions, one (2.5%) had proximal and middle lacrimal canalicular obstruction, and one (2.5%) had middle and distal lacrimal canalicular obstruction. Among 11 patients with ankylosing spondylitis, the onset of AU averaged 10.02 ± 9.34 years, with epiphora preceding uveitis by an average of 3.24 ± 3.42 years. Intraoperatively, 32 patients (80%) showed extensive adhesive LDO. Surgical procedures included laser canaliculoplasty with lacrimal stent intubation (55 eyes), dacryocystorhinostomy with lacrimal stent intubation (one eye), and retrograde canalicular trephination combined with dacryocystorhinostomy and lacrimal stent intubation (two eyes). Treatment outcomes showed a complete cure in 8 cases (20%), improvement in 21 cases (52.5%), and no effect in 11 cases (27.5%). The surgical success rates were significantly lower in uveitis patients compared to controls (*p* < 0.001), with extensive adhesions observed intraoperatively in 80% of uveitis cases.

**Conclusion:**

In patients with a history of AU, LDO predominantly affects the proximal lacrimal structures, often resulting in severe adhesions and poor surgical outcomes, with a cure rate of only 20%. The temporal link between epiphora and AU onset suggests shared pathophysiology. Targeted research is critical to clarify AU-LDO mechanisms and optimize prevention.

## Introduction

Epiphora often indicates an obstruction in the lacrimal duct system arising from diverse etiological factors (1). The drainage system is anatomically divided into proximal and distal segments. These obstructions, whether of congenital or acquired origin, may lead to chronic epiphora and mucopurulent discharge (2). Acquired lacrimal duct obstruction (LDO) can result from multiple causes, including severe viral conjunctivitis, chemical burns, chemotherapy, blepharitis, and prior trauma (3). Clinical observations have revealed that patients with a history of anterior uveitis (AU) frequently present significant adhesive obstructions in their lacrimal ducts, indicating a potential association between AU and these obstructions. Chronic inflammation in AU may lead to cytokine-driven fibrosis—mediated by factors such as IL-6 and TNF-*α*, potentially causing lacrimal duct epithelial damage and adhesion. This hypothesis is supported by elevated tear cytokine levels in uveitis patients (4). The management of such cases remains a significant therapeutic challenge, and surgical interventions often yield suboptimal outcomes. To investigate this relationship more thoroughly, a retrospective analysis was conducted on 40 patients who were diagnosed with and treated for LDO. This study aimed to examine and evaluate the clinical characteristics of patients with LDO and a history of AU, specifically focusing on those treated at the Third Medical Center of the Chinese People’s Liberation Army General Hospital.

## Subjects and method

### Participants

We retrospectively analyzed the medical records of 40 patients admitted to the Department of Ophthalmology of the Third Medical Center of the Chinese People’s Liberation Army General Hospital between January 2020 and December 2024. These patients presented with LDO and a history of AU. Patient selection was based on two primary inclusion criteria: (1) a verified history of AU and (2) a definitive diagnosis of LDO confirmed through slit-lamp examination and lacrimal passage flushing. Patients were excluded if they had lacrimal duct rupture, acute or chronic inflammation of the lacrimal canaliculus, acute or chronic dacryocystitis, lacrimal duct tumor, congenital lacrimal duct absence, or moderate to severe dry eye disease. For comparison, 40 control patients with LDO but no history of uveitis were randomly selected from the same period. This study followed the Declaration of Helsinki and was approved by the Ethics Committee of the Third Medical Center of the Chinese People’s Liberation Army General Hospital (KY2024-037).

### Operation technique and follow-up

Before surgery, all patients underwent a comprehensive ophthalmic examination, including lacrimal duct irrigation, exploration, and three-dimensional CT reconstruction of the lacrimal duct system. An experienced chief physician at our institution performed all the surgical procedures. Surgical approaches were selected based on preoperative evaluations and individual patient medical histories. The procedures included laser canaliculoplasty with lacrimal stent intubation; retrograde canalicular trephination combined with dacryocystorhinostomy and lacrimal stent intubation; dacryocystorhinostomy with lacrimal stent intubation; and canalicular dacryocystorhinostomy with canalicular obstruction resection and lacrimal stent intubation.

Following surgery, the patients were administered antibiotic eye drops, and their lacrimal passages were irrigated with a combination of antibiotics and glucocorticoids. Follow-up assessments were conducted at 3-, 6-, and 12-month intervals for these patients. The effectiveness of the surgical intervention was categorized as follows: “Cured” indicated complete resolution of epiphora symptoms, “Improved” signified a reduction in epiphora symptoms, and “Ineffective” denoted no change in epiphora symptoms.

## Items and methods

General demographic data, including sex, age, and other relevant characteristics, were collected. Clinical data collected included several parameters: the timing of the onset of epiphora and initial AU, ocular laterality of epiphora, systemic conditions associated with AU, anatomical location and extent of LDO, severity of obstruction, surgical intervention methodology, and therapeutic outcomes. A comparative analysis was performed between patients with a history of AU and a control cohort comprising individuals with LDO, but no history of uveitis during the same period. Demographic factors (age and sex), ocular involvement, anatomical characteristics of the LDO (location and extent), obstruction severity, and treatment efficacy were compared between the two groups.

### Statistical analysis

Statistical analysis of the collected data was performed using SPSS software (version 27.0; IBM Corp., Armonk, NY, United States). Descriptive statistical methods were employed, presenting measurement data as means, medians, and ranges. The count data were expressed as percentages (%). Following the assessment of data normality, t-tests were applied to normally distributed data, and non-parametric tests were applied to non-normally distributed data. The χ^2^ test was used for sample rate comparisons. A multiple logistic regression analysis was conducted to calculate odds ratios and 95% confidence intervals (CIs) to examine the associations between efficacy and various factors, including eye type, sex, age, obstructive segment, and history of AU. Statistical significance was set at a *p*-value of < 0.05.

## Result

### Demographic characteristics

Based on the established inclusion and exclusion criteria, 58 eyes of 40 patients diagnosed with AU were analyzed. The patient cohort had a mean age of 49.88 ± 10.18 years, with a median age of 52 years, ranging from 24 to 66 years. The control group, comprising individuals without a history of AU, had a mean age of 55.8 ± 13.64 years and a median age of 58.5 Â years, with ages ranging from 24 to 75 years. The AU group comprised 75% (*n* = 30) women and 25% (*n* = 10) men. Similarly, the control group included 65% (*n* = 26) women and 35% (*n* = 14) men.

### Clinical features

In the AU group, 9 cases (22.5%) occurred in the left eye, 13 cases (32.5%) in the right eye, and 18 cases (45.0%) affected both eyes. In the control group, 7 cases (17.5%) occurred in the left eye, 5 (12.5%) in the right eye, and 28 (70.0%) in both eyes. The AU group predominantly presented with proximal canalicular obstruction (<4 mm from the lacrimal punctum) and accounted for 32 patients (80%). These included 16 cases of isolated lacrimal punctum atresia, 6 cases of isolated proximal canaliculus obstruction, and 10 cases of lacrimal punctum atresia coexisting with lacrimal canaliculus obstruction. Furthermore, there was one case (2.5%) of middle lacrimal canalicular obstruction (>4 mm and <8 mm from the lacrimal punctum), two cases (5%) of distal canalicular obstruction (>8 mm from the lacrimal punctum), three cases (7.5%) of both proximal and distal canalicular obstruction, one case (2.5%) of proximal and middle canalicular obstruction, and one case (2.5%) of middle and distal canalicular obstruction. The control group primarily exhibited distal obstruction in 27 (67.5%) patients. In the AU group, HLA-B27 positivity was observed in 11 patients (four men and seven women), all of whom had concurrent ankylosing spondylitis. The mean time to initial AU onset was 10.02 ± 9.34 years (median: 6.5 years; range: 0.58–40 years). The average duration of tearing before uveitis diagnosis was 3.24 ± 3.42 years (median: 1.79 years; range: 0.08–15 years). The mean duration of epiphora in the control group was 5.20 ± 8.60 years (median: 3 years; range: 0.08–40 years). Surgical observations revealed extensive adhesive obstruction in 32 patients (80%) in the AU group, whereas eight patients (20%) did not exhibit such extensive adhesions. In contrast, no extensive adhesions were observed intraoperatively in the occluded segment of the control group (Table 1).

**Table 1:** Baseline characteristics of the case (*n* = 80).

Characteristics	Total (*n* = 80)	Anterior uveitis (*n* = 40)	Non anterior uveitis (*n* = 40)	*p* value
Age, mean ± SD, *n* (%)	52.84 ± 12.32	49.88 ± 10.18 (50)	55.80 ± 13.64 (50)	0.017
Sex, *n* (%)				0.329
Male	24 (30)	10 (25)	14 (35)	
Female	56 (70)	30 (75)	26 (65)	
Eye, *n* (%)				0.050
Right	18 (22.5)	13 (32.5)	5 (12.5)	
Left	16 (20)	9 (22.5)	7 (17.5)	
Both	46 (57.5)	18 (45)	28 (70)	
Blocking segment, *n* (%)				0.000
Proximal part	39 (48.8)	32 (80)	7 (17.5)	
Middle part	4 (5)	1 (2.5)	3 (7.5)	
Distal part	29 (36.3)	2 (5)	27 (67.5)	
Proximal and middle part	4 (5)	3 (7.5)	1 (2.5)	
Proximal and distal part	1 (1.3)	1 (2.5)	0 (0)	
Middle and distal part	3 (3,8)	1 (2.5)	2 (5)	
Extensive adhesion, *n* (%)				0.000
Yes	32 (40)	32 (80)	0 (0)	
No	48 (60)	8 (20)	40 (100)	
Curative effect				0.000
Cured	40 (50)	8 (20)	32 (80)	
Improval	28 (35)	21 (52.5)	7 (17.5)	
Not cured	12 (15)	11 (27.5)	1 (2.5)	

*T*-test was used to compare continuous variables. χ^2^ test was used to compare the sample rate.

### Surgical techniques and clinical outcomes

Of the 39 patients in the AU group, all underwent laser canaliculoplasty with lacrimal stent intubation. Among them, two patients required retrograde canalicular trephination combined with dacryocystorhinostomy and lacrimal stent intubation, while the contralateral eye underwent lacrimal duct laser plasty combined with lacrimal stent intubation. Moreover, another patient underwent a dacryocystorhinostomy combined with lacrimal stent intubation. Among the 38 patients in the control group, 38 underwent laser canaliculoplasty with lacrimal stent intubation. Additionally, two patients underwent canalicular dacryocystorhinostomy, including canalicular obstruction resection and lacrimal stent intubation.

Patients underwent follow-up examinations at 3, 6, and 12 months postoperatively. In the AU group, the treatment outcomes were as follows: eight patients (20%) achieved complete recovery, 21 patients (52.5%) showed improvement, and 11 patients (27.5%) remained uncured. In the control group, the observed outcomes were as follows: 32 patients (80%) achieved complete recovery, 7 (17.5%) demonstrated improvement, and 1 (2.5%) remained uncured (Table 1).

### Analysis of treatment efficacy and associated factors

**Figure 1:**
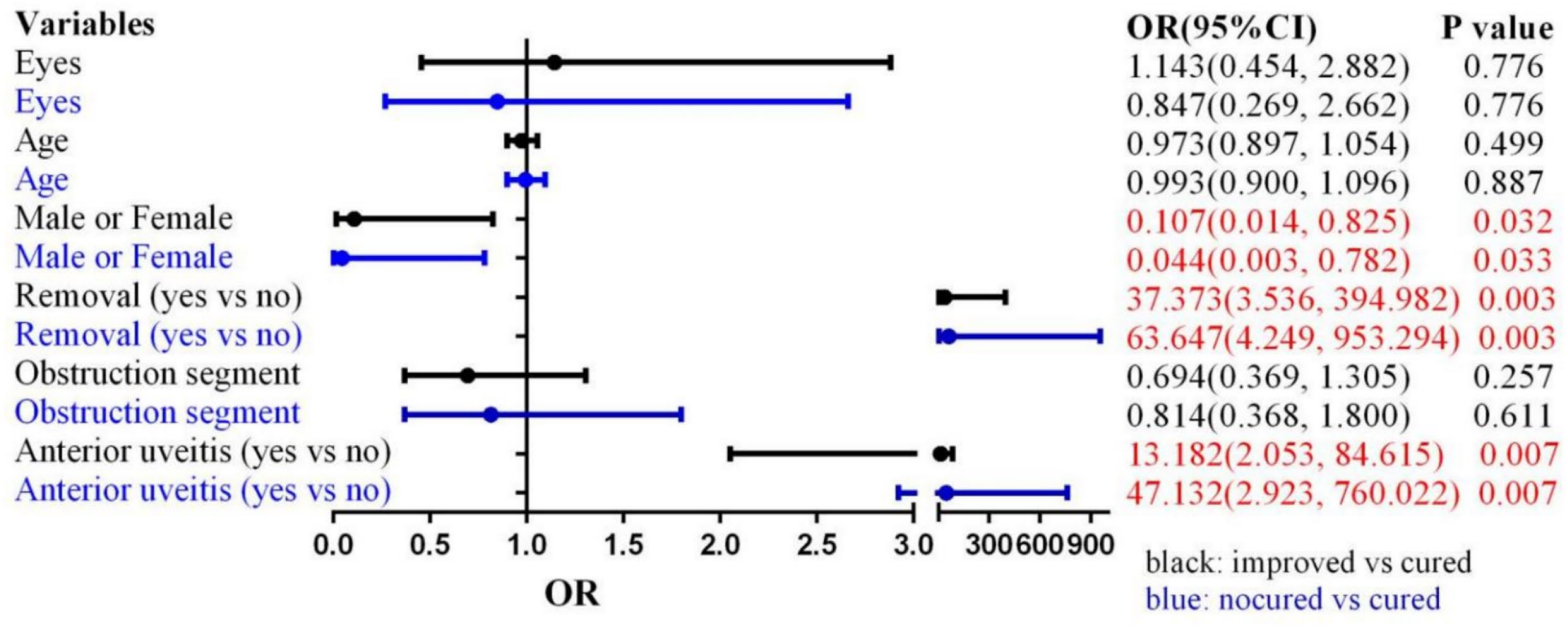
Estimated ORs (95% CI) in curative effect and involved factors by multivariate logistic regression analysis. OR: Odds Ratio CI: Confidence Interval.

Analysis of the influence of AU on postoperative outcomes in patients with LDO revealed that AU independently exerted a significant negative effect on surgical results (*p* = 0.000) (Table 2). Subsequent multivariate analysis confirmed that AU had a detrimental effect on LDO outcomes (effective vs. cured: 13.182 (2.053, 84.615), *p* = 0.007; ineffective vs. cured: 47.132 (2.923, 760.022), *p* = 0.007) (Figure 1).

**Table 2:** Adjusted 95%CI for curative effect and involved factors.

Baseline variable adjusted for	Treatment effectiveness	OR (95% CI)	*P* value
Crude model		29.733	0.000
Model A	Improved VS cured	14.237 (3.771, 53.747)	0.000
	Not cured VS cured	55.844 (5.534, 563.504)	0.001
Model B	Improved VS cured	13.182 (2.053, 84.615)	0.007
	Not cured VS cured	47.132(2.923, 760.022)	0.007

Crude model: adjusted no factors associated with anterior uveitis (yes or no) and curative effect. Model A: adjusted for sex, age. Model B: adjusted for model A plus eye type, blocking segment, history of anterior uveitis (yes or no), treatment effectiveness (improved, cured, not cured).

## Discussion

The lacrimal drainage system consists of the lacrimal puncta, upper and lower canaliculi, common lacrimal duct, lacrimal sac, and nasolacrimal duct (2). Stenosis or obstruction of any segment of the system can result in varying degrees of epiphora. Lacrimal punctal and canalicular obstruction, alternatively termed proximal LDO (5), is characterized by a membranous obstruction anterior to the lacrimal sac and includes various obstructive conditions, such as lacrimal puncta stenosis, canalicular obstruction, and common canalicular obstruction (6). Lacrimal canalicular obstruction was further classified as proximal lacrimal canalicular obstruction (<4 mm from the lacrimal punctum), middle lacrimal canalicular obstruction (≥4 mm and ≤8 mm from the lacrimal punctum), or distal lacrimal canalicular obstruction (>8 mm from the lacrimal punctum) (1). The etiology of lacrimal punctal and canalicular obstruction is complex and involves multiple factors, including bacterial and viral infections, ocular surface diseases, medication use, chemical burns, and cicatricial conditions (6).

Lacrimal punctal obstruction, a common cause of epiphora with reported incidence rates between 8 and 54.3% (1), manifests in either congenital or acquired forms. Congenital absence of the lacrimal puncta typically results from a developmental failure of the lacrimal canaliculus to bud from the superior portion of the solid lacrimal cord during the embryonic stage of 18–24 mm (7). The pathogenesis of acquired lacrimal puncta obstruction involves multiple factors, including age-related degenerative changes, such as eyelid relaxation, displacement, and ectropion (8). Contributing factors encompass chronic blepharitis; viral and bacterial infections, including chlamydia, herpes simplex virus, actinomycetes, and human papillomavirus (9, 10); and systemic and topical medications, including 5-fluorouracil, docetaxel, and paclitaxel (11). Furthermore, various systemic conditions, including enteropathic acrodermatitis, porphyria tarda, Stevens–Johnson syndrome, ocular cicatricial pemphigoid, and graft-versus-host disease, may lead to lacrimal puncta obstruction (11).

Canalicular obstruction constitutes 16–25% of epiphora cases (7) and manifests as either a congenital or acquired condition. Unilateral or bilateral canalicular hypoplasia occurs in 4% of patients and may present in isolation or alongside other developmental abnormalities (12). Various conditions can result in canalicular obstruction, including conjunctivitis, chronic blepharitis, cicatricial pemphigoid, Stevens–Johnson syndrome, and lichen planus. In addition, chemical and thermal burns, traumatic lacerations, skin cancer, papilloma, and radiotherapy can affect the canalicular system, leading to obstruction (11). Furthermore, several medical interventions can lead to canalicular stenosis or obstruction, including verteporfin photodynamic therapy for choroidal neovascularization (13), local anti-glaucoma medications (14), and chemotherapeutic agents, such as 5-fluorouracil, docetaxel, mitomycin C, and pemetrexed (15, 16). Additional contributing factors to LDO include dislodgment of lacrimal plugs (11) and iatrogenic trauma.

This study demonstrated that patients with a history of AU predominantly displayed proximal obstruction, including lacrimal punctal atresia and/or proximal LDO. By contrast, the control group, without a history of AU, primarily presented with distal LDO, with this difference between the groups showing statistical significance. LDO in patients with AU occurs in the segment anterior to the lacrimal sac. However, the relationship between AU and LDO has not been reported in existing literature and is worthy of further investigation.

Uveitis, characterized by inflammation of one or more components of the uveal tract (17), is a significant cause of vision loss worldwide (18). This condition accounts for 10% of blind cases in industrialized nations (19) and approximately 25% of irreversible blindness cases in developing countries (20). With an estimated annual incidence of 17 cases per 100,000 people (19, 21), uveitis predominantly affects individuals aged 20–50 years (22). The condition is classified into infectious and non-infectious uveitis based on etiology, with non-infectious cases occurring more frequently.

AU represents the most common manifestation of uveitis (23). This condition encompasses three subtypes: iritis, iridocyclitis, and anterior ciliary body inflammation. Research data demonstrate that AU accounts for 30–73% of all uveitis cases, with associated blindness rates ranging from 0.6 to 11% (16, 19). Common clinical presentations include ocular redness, photophobia, lacrimation, and impaired vision. Visual deterioration may result from inflammatory processes and subsequent complications, such as macular edema, glaucoma, and cataract formation. This condition primarily affects individuals aged 20–40 years (64%) and is less prevalent in those aged >60 years (10%) (21).

The current study found that patients with a history of LDO and AU had a mean age of 49.88 years (range: 24–66 years), which is consistent with existing literature. In Western nations, the predominant forms of AU are HLA-B27 (+) uveitis, Fuchs’ heterochromic iridocyclitis, and herpes zoster uveitis (24). HLA-B27-related AU and syphilitic uveitis occur more frequently in men, whereas women more frequently experience chronic AU associated with juvenile idiopathic arthritis, multiple sclerosis, granulomatous disease, and Vogt–Koyanagi–Harada disease. Evidence suggests that non-infectious AU occurs more frequently in women (23). Moreover, the incidence of LDO is higher in women. Female predominance was notable in uveitis-associated lacrimal obstruction (75% women vs. 25% men), consistent with the higher prevalence of chronic AU in women.

This study investigated the initial onset of AU in 40 patients with a history of LDO and AU. The mean onset interval was 10.02 years, with patients experiencing tearing for an average of 5.20 years before uveitis diagnosis. All patients with a history of AU were HLA-B27 positive, and 11 were diagnosed with ankylosing spondylitis (four men and seven women). Intraoperative examination revealed extensive adhesive obstruction of the lacrimal duct in 32 cases, whereas eight cases exhibited no extensive adhesive obstruction. Statistical analyses demonstrated significant differences between the experimental and control groups, indicating that patients with a history of AU predominantly present with extensive adhesions within their lacrimal ducts. Furthermore, AU may cause tearing symptoms that mimic inflammatory recurrence, potentially leading to misinterpretation of these symptoms and a consequent delay in lacrimal duct examination. As epiphora becomes apparent, LDO frequently progresses to a severe and extensive stage.

The literature presents a comprehensive treatment framework for proximal LDO. For patients with LDO, established procedures include three-snip punctoplasty or punctal dilation in conjunction with either single or double lacrimal duct stents, such as the Mini-Monoka, O’Donoghue, Crawford tube, or Ritleng tubes (11). Recent studies have introduced canaliculotomy as a viable treatment option for lacrimal punctal and canalicular atresia (25). Patients with severe lacrimal canalicular obstruction can be classified into three categories: (1) patients with proximal lacrimal canalicular obstruction, who require dacryocystorhinostomy with retrograde catheterization (26); (2) patients with middle lacrimal canalicular obstruction, for whom treatment options include retrograde catheterization via dacryocystorhinostomy, insertion of an LJT/StopLossTM tube combined with conjunctival dacryocystorhinostomy, or utilization of a LacriJetTM tube with a trephine; and (3) patients with distal lacrimal canalicular obstruction, for whom the standard approach is conjunctival dacryocystorhinostomy with an LJT/StopLoss tube or trephine surgery combined with artificial lacrimal duct insertion.

Among patients with a history of AU, the treatment outcomes were as follows: 8 achieved complete cure, 21 demonstrated improvement, and 11 showed no improvement. Multivariate logistic regression analysis examining the correlation between treatment outcomes and AU revealed that the surgical group had significantly poorer results than those of the control group.

Research work indicates that uveitis is strongly associated with inflammatory and immune cytokines, including tumor necrosis factor, interleukin 1 (IL-1), IL-2, IL-6, IL-8, and interferon gamma (IFN-*γ*) (27). Patients with uveitis have elevated levels of cytokines and chemokines in the serum and aqueous humor (28, 29). Additionally, tear samples from these patients show significantly higher concentrations of IL-1RA, IL-8/CXCL8, fractalkine/CX3CL1, IP-10/CXCL10, vascular endothelial growth factor (VEGF), and TGF-β2 compared to those of healthy individuals (4). A total of 10 proinflammatory cytokines, including matrix metalloproteinase 9, serine protease E1, IL-6, hepatocyte growth factor, VEGFA, VEGFR2, platelet endothelial cell adhesion molecule 1, C-reactive protein, chemokine ligand 2, and platelet-derived growth factor-AA (PDGF-AA), are significantly upregulated in patients with nasolacrimal duct obstruction (nasoLDO) (30). Wang et al.’s preliminary investigation of cytokines in the tears of patients with LDO indicated that inflammatory factors play a fundamental role in disease onset and progression (31). Elevated concentrations of IFN-*α*2a, IFN-*β*, IFN-*γ*, IL-17A, IL-6, IL-8, tumor necrosis factor-*α*, and VEGF-A may serve as predictive markers for lacrimal duct obstructive disorders. Considering the critical role of cytokines in the pathogenesis of duct obstruction, evidence suggests an association between AU and this condition.

Inspired by those studies and considering the significant role of cytokines in the pathogenesis of LDO, we believe that there is a tight association between AU and LDO. Elevated levels of IL-6 and TNF-α in uveitis patients may promote fibroblast activation and collagen deposition, leading to lacrimal duct fibrosis. This finding aligns with our finding of extensive adhesions in 80% of surgical cases, suggesting chronic inflammation as a key driver of obstruction. This condition primarily affects women, with the obstruction most commonly occurring anterior to the lacrimal sac, specifically involving lacrimal punctal atresia and proximal lacrimal canalicular blockage. These conditions frequently result in substantial adhesion. Clinicians should maintain a high index of suspicion for proximal lacrimal obstruction in uveitis patients, particularly among women presenting with refractory epiphora.

To establish a definitive correlation between AU and duct obstruction, extensive data collection and multicenter follow-up studies are required. This research necessitates large-scale sample analysis, histopathological examination, and investigation of cytokine concentration variations in affected patients. Such a systematic investigation is critical for studying the pathogenic mechanisms underlying these conditions and for establishing a foundation to examine the relationship between AU and duct obstruction, ultimately facilitating the development of effective preventive measures.

### Limitations

Limitations include the single-center design and the lack of longitudinal cytokine profiling. Future multicenter studies with serial tear cytokine measurements are needed to validate the inflammation-fibrosis hypothesis.

## Conclusion

In this study, we summarize clinical characteristics of LDO patients with prior AU, revealing distinctive features such as lacrimal punctal atresia, proximal canalicular obstruction, and extensive adhesive obstructions that present significant therapeutic challenges. We explored the relationship between AU and LDO from multiple perspectives, including statistical analysis and the study of inflammatory and immune cytokines. Our findings indicate that clinicians should maintain a high index of suspicion for proximal lacrimal obstruction in uveitis patients, particularly in women presenting with refractory epiphora. Future research will focus on establishing a definitive correlation using a large sample size from multiple centers.

## Data Availability

All relevant data are within the paper and its supporting information files. Research data supporting this publication are available from the NN repository located at: www.NNN.org/download/.
